# Sodium butyrate facilitates CRHR2 expression to alleviate HPA axis hyperactivity in autism-like rats induced by prenatal lipopolysaccharides through histone deacetylase inhibition

**DOI:** 10.1128/msystems.00415-23

**Published:** 2023-06-26

**Authors:** Xinyuan Wang, Zhujun Sun, Ting Yang, Fang Lin, Shasha Ye, Junyan Yan, Tingyu Li, Jie Chen

**Affiliations:** 1 Chongqing Key Laboratory of Childhood Nutrition and Health, Children’s Hospital of Chongqing Medical University, Chongqing, China; 2 Ministry of Education Key Laboratory of Child Development and Disorders, National Clinical Research Center for Child Health and Disorders, Chongqing, China; Istanbul Medipol University School of Medicine, Istanbul, Turkey

**Keywords:** short chain fatty acids, glucocorticoid, HPA axis, autism, social behavior, histone deacetylases

## Abstract

Short-chain fatty acids (SCFAs, especially butyric acid) have been demonstrated to play a promising role in the development of autism spectrum disorders (ASD). Recently, the hypothalamic–pituitary–adrenal (HPA) axis is also suggested to increase the risk of ASD. However, the mechanism underlying SCFAs and HPA axis in ASD development remains unknown. Here, we show that children with ASD exhibited lower SCFA concentrations and higher cortisol levels, which were recaptured in prenatal lipopolysaccharide (LPS)-exposed rat model of ASD. These offspring also showed decreased SCFA-producing bacteria and histone acetylation activity as well as impaired corticotropin-releasing hormone receptor 2 (CRHR2) expression. Sodium butyrate (NaB), which can act as histone deacetylases inhibitors, significantly increased histone acetylation at the CRHR2 promoter *in vitro* and normalized the corticosterone as well as CRHR2 expression level *in vivo*. Behavioral assays indicated ameliorative effects of NaB on anxiety and social deficit in LPS-exposed offspring. Our results imply that NaB treatment can improve ASD-like symptoms via epigenetic regulation of the HPA axis in offspring; thus, it may provide new insight into the SCFA treatment of neurodevelopmental disorders like ASD.

**IMPORTANCE:**

Growing evidence suggests that microbiota can affect brain function and behavior through the “microbiome–gut–brain’’ axis, but its mechanism remains poorly understood. Here, we show that both children with autism and LPS-exposed rat model of autism exhibited lower SCFA concentrations and overactivation of HPA axis. SCFA-producing bacteria, *Lactobacillus*, might be the key differential microbiota between the control and LPS-exposed offspring. Interestingly, NaB treatment contributed to the regulation of HPA axis (such as corticosterone as well as CRHR2) and improvement of anxiety and social deficit behaviors in LPS-exposed offspring. The potential underlying mechanism of the ameliorative effect of NaB may be mediated via increasing histone acetylation to the CRHR2 promoter. These results enhance our understanding of the relationship between the SCFAs and the HPA axis in the development of ASD. And gut microbiota-derived SCFAs may serve as a potential therapeutic agent to neurodevelopmental disorders like ASD.

## INTRODUCTION

Autism spectrum disorder (ASD) is a neurodevelopmental disorder characterized by impairments in social communication, repetitive and stereotyped behavior, and restricted interests (1, 2). The etiology of ASD is rather complicated, in part due to the substantial genetic heterogeneity, the complexity of gene–environment interactions, and the variability in phenotypic presentation (1, 3). Maternal immune activation (MIA) has been increasingly implicated in neurodevelopmental outcomes associated with ASD, as large epidemiological studies have shown that maternal infection during pregnancy may be associated with the occurrence probability of ASD in offspring (4). Importantly, prenatal lipopolysaccharide (LPS) treatment is a well-established model for ASD-like social behavioral deficits and neurocognitive impairments (5, 6). Accumulating studies in ASD research fields indicate that microbiota dysbiosis of the gastrointestinal system and its metabolites contribute to the development of ASD (7). Short-chain fatty acids (SCFAs), the gut microbial fermentation of dietary fiber, are found altered in ASD children (8). It has been noted that SCFAs can reach the brain owing to the abundant expression of monocarboxylate transporters (9, 10). Besides, SCFAs might influence psychological functioning via interactions with G protein-coupled receptors (GPCRs) or histone deacetylases (HDACs) and exert their effects on the brain via hormonal and immune pathways as well as vague nerve signaling (11). Growing evidence about the involvement of SCFAs has been implicated in immune dysfunction, cognitive disorder, and behavior deficits (12). However, it is still unclear how the altered SCFA levels in the gut affect these behaviors via the microbiota–brain axis.

The hypothalamic–pituitary–adrenal (HPA) axis, as part of the microbiota–brain axis, including the paraventricular nucleus of the hypothalamus (PVN) releases corticotropin-releasing hormone (CRH), anterior pituitary secretes adrenocorticotropic hormone, and adrenal glands stimulate the secretion of cortisol (13). When the host’s cortisol is overloaded, a negative feedback mechanism will be activated to downregulate the stress response (14). Glucocorticoids (GCs, corticosterone in rodent, cortisol in human) are considered to be key elements in the HPA axis response and exert extensive effects in the center neural system as well as the periphery system (15). A clinical trial demonstrated that SCFAs significantly attenuated the cortisol response to psychosocial stress (16). Besides, microbiota deficiency exacerbates HPA activity in response to stressors, also suggesting that the gut microbiome participates in regulating the HPA axis (17, 18). Recently, research found microbiome could affect social behaviors through mediating stress responses neurons in brain, and downregulated CRH neurons could reverse social impairments in antibiotic-treated mice (19). However, microbial molecules that are responsible for modulating social activity are still unknown.

Considering that SCFAs can impact the functioning of the HPA axis and both of them play an important role in social behavior, we investigated the potential mechanism behind them. First, we investigated the abnormal changes in SCFAs composition and GC levels in ASD children and LPS-exposed ASD rats. Then, we assessed differences in the expression levels of histone acetylation as well as CRH and its receptor *in vivo* and *in vitro*. Finally, we observed the benefits of sodium butyrate (NaB) intervention on social communication of LPS-exposed rats. Our study reflects a mechanistic investigation on how butyrate leads to the rescue of behavioral deficits, which is accompanied by normalization of HPA axis and histone acetylation in LPS-exposed ASD rats.

## RESULTS

### SCFAs and GC levels were altered in children with ASD and LPS-exposed offspring rats

**Fig 1:**
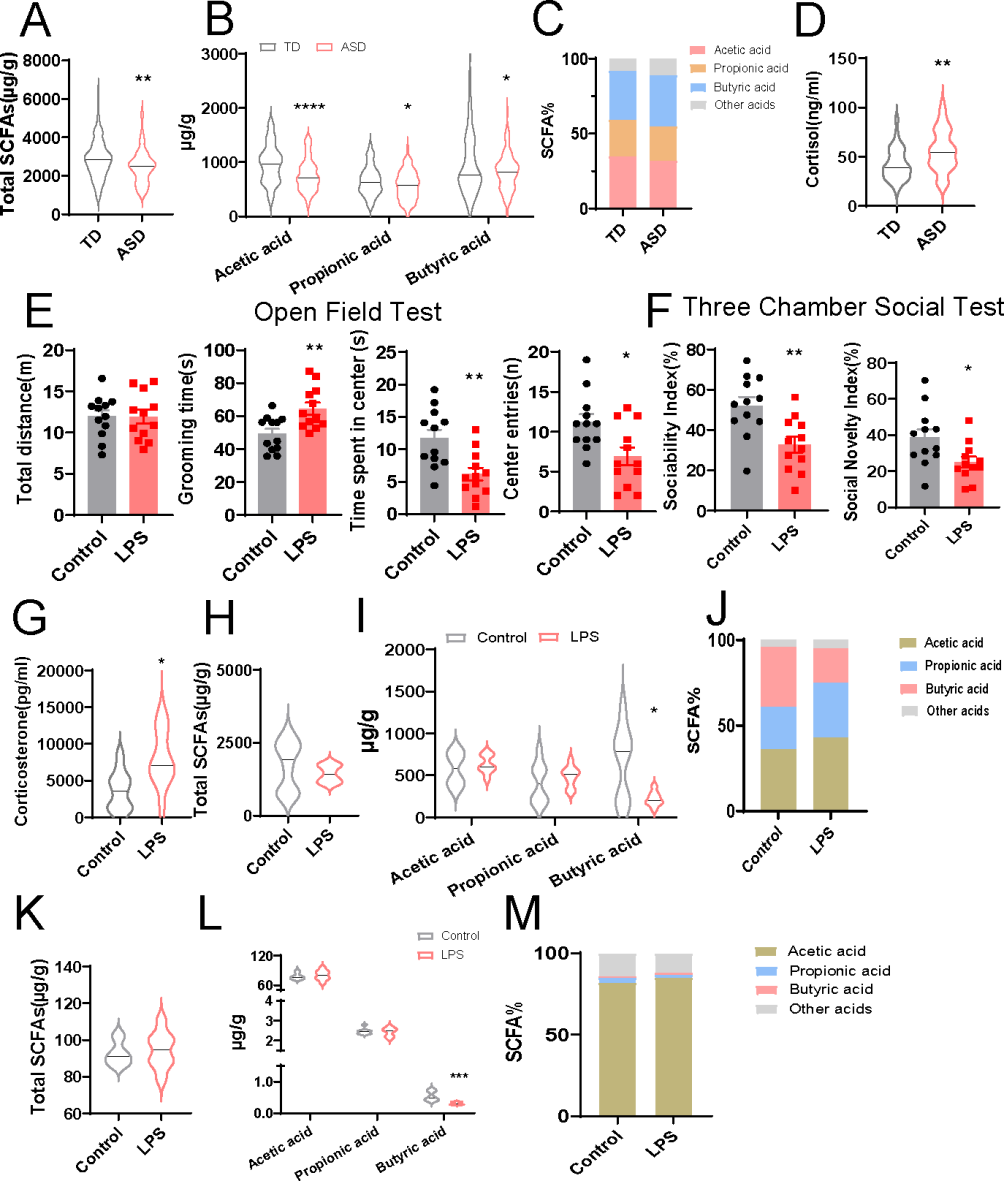
Alterations of the SCFAs and HPA axis hyperactivity in children with ASD and LPS-exposed offspring rats. (A and B) Total SCFAs levels (A), acetic, propionic, and butyric acid levels (B) in feces of TD children and children with ASD (*n* = 83–84 per group, two-tailed t-test). (C) Percentages of acetic, propionic, butyric, and other acids in feces of TD children and children with ASD (*n* = 83–84 per group, χ^2^ test). (D) Cortisol level in plasma of TD children and children with ASD (*n* = 33–34 per group, two-tailed t-test). (E) Total distance traveled, the time spent in grooming, the time spent in center area, and number of center entries during the open field test between the control and LPS-exposed offspring rats (*n* = 12 per group, two-tailed t-test). (F) Sociability or social novelty index during the three-chamber social test between the control and LPS-exposed offspring rats (*n* = 12 per group, two-tailed *t*-test). (G) Corticosterone level in serum of control and LPS-exposed offspring rats (*n* = 12 per group, two-tailed t-test). (H and I) Total SCFAs levels (H), acetic, propionic, and butyric acid levels (I) in feces of control and LPS-exposed offspring rats (*n* = 8 per group, two-tailed *t*-test). (J) Percentages of acetic, propionic, butyric, and other acids in feces of control and LPS-exposed offspring rats (*n* = 8 per group, χ^2^ test). (K and L) Total SCFAs levels (K), acetic, propionic, and butyric acid levels (L) in brain tissues of control and LPS-exposed offspring rats (*n* = 8 per group, two-tailed t-test). (M) Percentages of acetic, propionic, butyric, and other acids in brain tissues of control and LPS-exposed offspring rats (*n* = 8 per group, χ^2^ test). Data are expressed as mean ± SEM. ****P < 0.0001, ***P < 0.001, **P < 0.01, *P < 0.05.

In this study, we analyzed fecal samples from a total of 167 participants, including 84 autistic children and 83 typically developing (TD) children (Table 1). No statistically significant differences were observed in the gender ratio of the two groups. The average age of children in the ASD group and TD group at the time of sample collection was 4.52 (range, 3–6) and 4.35 (range, 2–6) years old, respectively (Table 1). We compared the concentrations of total SCFAs, including acetic acid, propionic acid, and butyric acid from the fecal samples between the ASD and TD groups (Table S1). As shown in Fig. 1A and B, the total amount of SCFAs, the levels of acetic, propionic, and butyric acid were significantly lower in the ASD group compared with those in the TD group. However, the composition proportion of acetic, propionic, butyric, and other acids (isovaleric, valeric, hexanoic, heptanoic, octanoic, nonanoic, and decanoic acid) was identical between them (Fig. 1C). Next, we compared the plasma cortisol concentrations in this cohort. Consistent with previous research (20), the cortisol levels in ASD children (56.35 ± 3.631 ng/mL) were markedly higher than that in the TD group (42.38 ± 2.816 ng/mL, Fig. 1D). These clinical data suggest that ASD children have an abnormally decreased SCFAs concentrations and increased cortisol levels.

**TABLE 1:** Demographic and clinical characteristics of participants

	TD	ASD	*P*-value
Numbers of participants	83	84	
Age (years), mean ± SD	4.52 ± 0.78	4.35 ± 0.86	0.1770
Sex (male/female)	55/28	67/17	0.0564
ABC^*a*^			
Sensory	-	8.71 ± 0.68	
Social withdrawal	-	14.59 ± 0.85	
Stereotypic behavior	-	9.19 ± 0.92	
Inappropriate speech	-	12.47 ± 0.79	
Laggard daily living ability	-	11.01 ± 0.60	
Total ABC scores	-	55.98 ± 3.27	
SRS^*b*^			
Social awareness	-	11.52 ± 0.33	
Social cognition	-	17.83 ± 0.51	
Social communication	-	33.43 ± 1.00	
Social motivation	-	15.10 ± 0.61	
Autistic mannerisms	-	13.65 ± 0.77	
Total SRS scores	-	91.92 ± 2.61	
CARS^*c*^ total scores	-	37.69 ± 0.69	
ADOS^*d*^ total scores	-	17.13 ± 0.52	

^*a*^ ABC: Autism Behavior Checklist.

^*b*^ SRS: Social Responsiveness Scale.

^*c*^ CARS: Childhood Autism Rating Scale.

^*d*^ ADOS: Autism Diagnostic Observation Schedule.

As well-studied, LPS-exposed offspring rats displayed stereotyped and anxiety behavior, e.g., longer grooming time, shorter center duration time and fewer center entries (Fig. 1E), impaired sociability, and decreased preference for social novelty compared to control (Fig. 1F). Similar with the alterations in SCFAs and cortisol above, LPS-exposed rats was found with higher corticosterone in serum and lower butyric acid in feces compared with the control group (Fig. 1G through I). Acetic and propionic acid levels in feces were detected with no significant differences between them (Fig. 1I). Considering the SCFAs changes in feces, we also measured the SCFAs concentration in the brain for further study. Interestingly, though the content was such little, the butyric acid was also lower in the LPS-exposed rats compared with that of the control group (Fig. 1L). Additionally, the total SCFAs level and their composition proportion were still unchanged both in the feces and the brain tissues of the two groups (Fig. 1H, J, K, and M). Overall, these results imply that altered SCFAs concentration and corticosterone may play a critical role in ASD-like symptoms of prenatal LPS-exposed offspring.

### Differential microbial community structures and decreased SCFA-producing microbiota in LPS-exposed offspring

**Fig 2:**
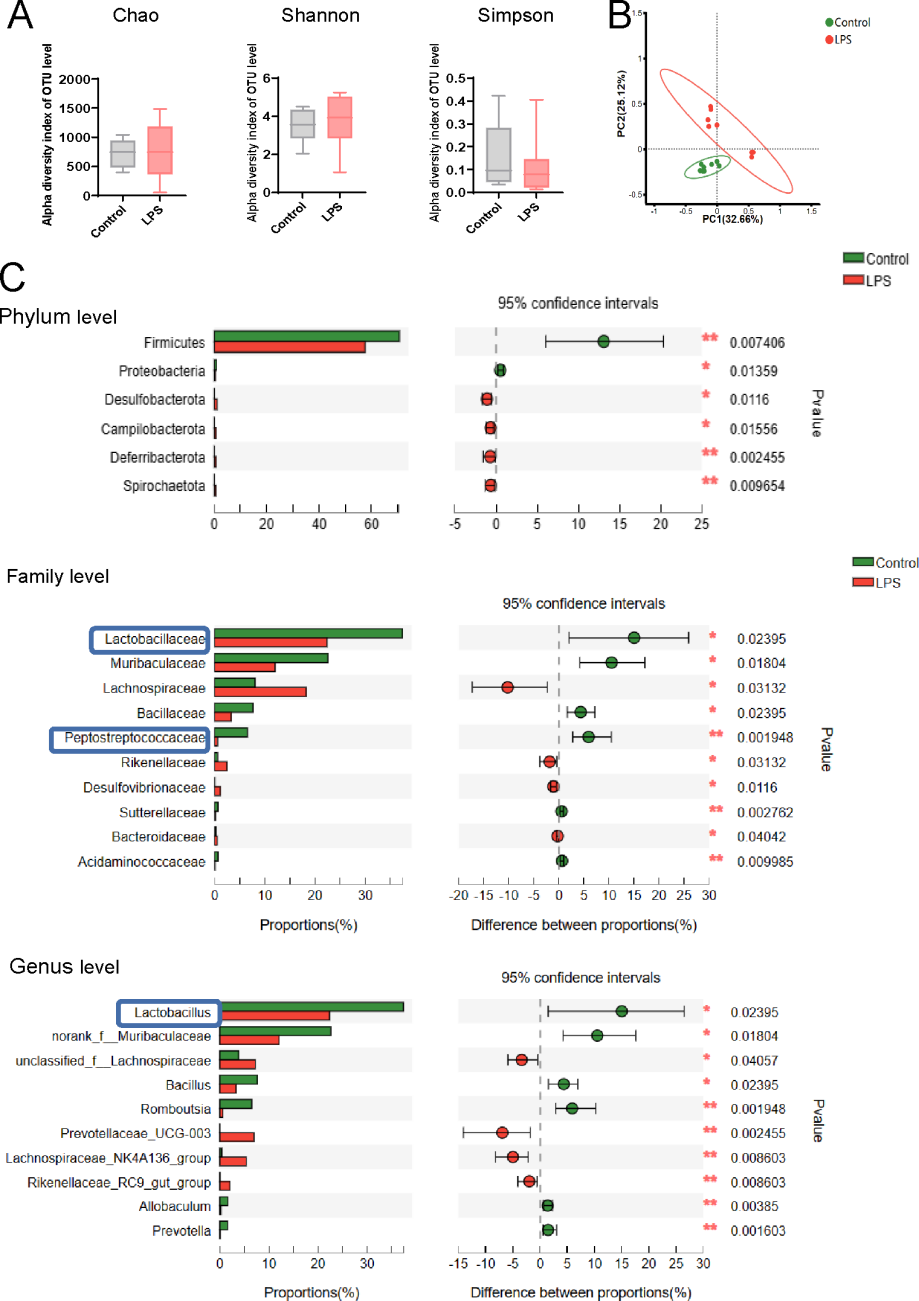
Changes in the gut microbiota composition in LPS-exposed offspring rats. (A) Alpha diversity of the gut microbiota (Chao, Shannon, and Simpson indices) between the control and LPS-exposed offspring rats at the amplicon sequence variant (ASV) level (*n* = 8 per group, Wilcoxon rank sum test followed by Tukey post hoc test). (B) PCoA plots of the microbiota composition between the control and LPS-exposed offspring rats at the ASV level (*n* = 8 per group). (C) Comparison of the dominant microbiota at the phylum, family, and genus levels between the control and LPS-exposed offspring rats. SCFA-producing bacteria are marked with blue frames. (**P < 0.01, *P < 0.05, *n* = 8 per group, Wilcoxon rank sum test followed by Tukey post hoc test.)

We then compared the gut microbial composition using 16S ribosomal RNA gene sequencing to detect species differential abundance in fecal samples of LPS-exposed offspring. No statistically significant differences were found in α diversity (Chao, Shannon, and Simpson diversity indices) between the two groups (Fig. 2A). However, principal coordinate analysis (PCoA) plots exhibited conspicuous differences in microbial community structures between the control and LPS-exposed offspring rats (Fig. 2B). To further identify the key microbiota, the abundance changes in the gut microbiota were evaluated. The dominant significantly differential microbiota composition between the control and LPS-exposed offspring rats at the phylum, family, and genus levels was shown in Fig. 2C. For example, the abundance of *Firmicutes* was significantly lower in LPS-exposed offspring than in the control group at phylum level. Then the abundance of *Lactobacillaceae, Muribaculaceae, Bacillaceae,* and *Peptostreptococcaceae* were decreased and *Lachnospiraceae* was increased in LPS-exposed offspring at the family level. We also detected a lower abundance of SCFA-producing bacteria, *Lactobacillus,* at the genus level in LPS-exposed rats than in the control group (21) (Fig. 2C). These results above showed the different microbial communities as well as the lower abundance of SCFA-producing bacteria in LPS-exposed rats.

### Histone acetylation decreased and CRHR2 impaired in LPS-exposed offspring

**Fig 3:**
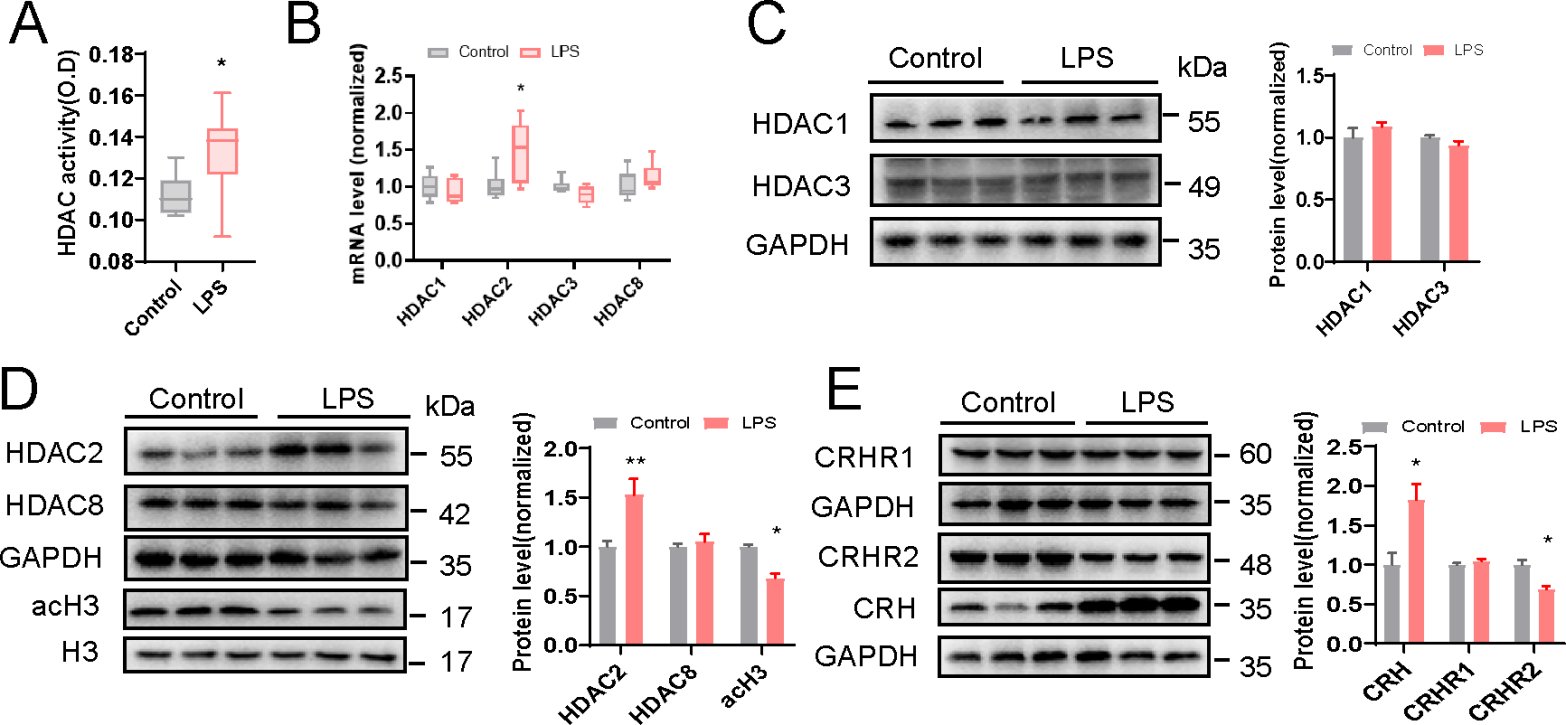
Downregulated histone acetylation and impaired CRH or its receptor expression in the hypothalamus of LPS-exposed offspring. (A) HDAC activity in the hypothalamus of control and LPS-exposed offspring rats was determined using HDAC Activity Colorimetric Assay Kit (*n* = 9 per group, two-tailed *t*-test). (B) HDAC1, HDAC2, HDAC3, and HDAC8 mRNA expression in the hypothalamus of control and LPS-exposed offspring rats, as detected using qPCR and normalized to GAPDH (*n* = 6 per group, two-tailed *t*-test). (C and D) Western blot and quantification analyses of HDAC1, HDAC2, HDAC3, HDAC8, and acH3 in the hypothalamus of control and LPS-exposed offspring rats (*n* = 3 per group, two-tailed *t*-test). (E) Western blot and quantification analyses of CRH, CRHR1, and CRHR2 in the hypothalamus of control and LPS-exposed offspring rats (*n* = 3 per group, two-tailed *t*-test). Data are expressed as mean ± SEM. **P < 0.01, *P < 0.05.

The hypothalamus of LPS-exposed offspring rats showed a significant increase in global HDAC activity when compared with control rats (Fig. 3A). Furthermore, we detected class I HDACs subtypes (HDAC1, HDAC2, HDAC3, and HDAC8) expression levels in the hypothalamus between the two groups. Quantitative PCR analyses indicated that the level of HDAC2 mRNA expression was significantly higher in the hypothalamus lysate from LPS-exposed offspring, while the levels of HDAC1, HDAC3, and HDAC8 were largely unchanged (Fig. 3B). Similarly, western blot assays showed the protein expression level of HDAC2 in the hypothalamus was also significantly higher in LPS-exposed offspring rats (Fig. 3D). Additionally, the ratio of acH3 to H3 in LPS-exposed offspring was significantly reduced compared to that of the control group (Fig. 3D). These results demonstrate the elevation of histone deacetylation levels in the hypothalamus of LPS-exposed offspring.

Given that histone acetylation regulates gene expression and butyric acid concentration was found decreased in both the brain and feces of LPS-exposed offspring, we also detected the expression levels of CRH and its receptors in hypothalamus for further study. Compared with the control group, LPS-exposed offspring exhibited increased CRH and decreased CRHR2 expression levels (Fig. 3E). Together, these data suggest that LPS exposure during gestation induces CRH regulation abnormalities accompanied by decreased histone acetylation in offspring.

### NaB treatment reversed CRHR2 decrease induced by dexamethasone in PC12 cells

**Fig 4:**
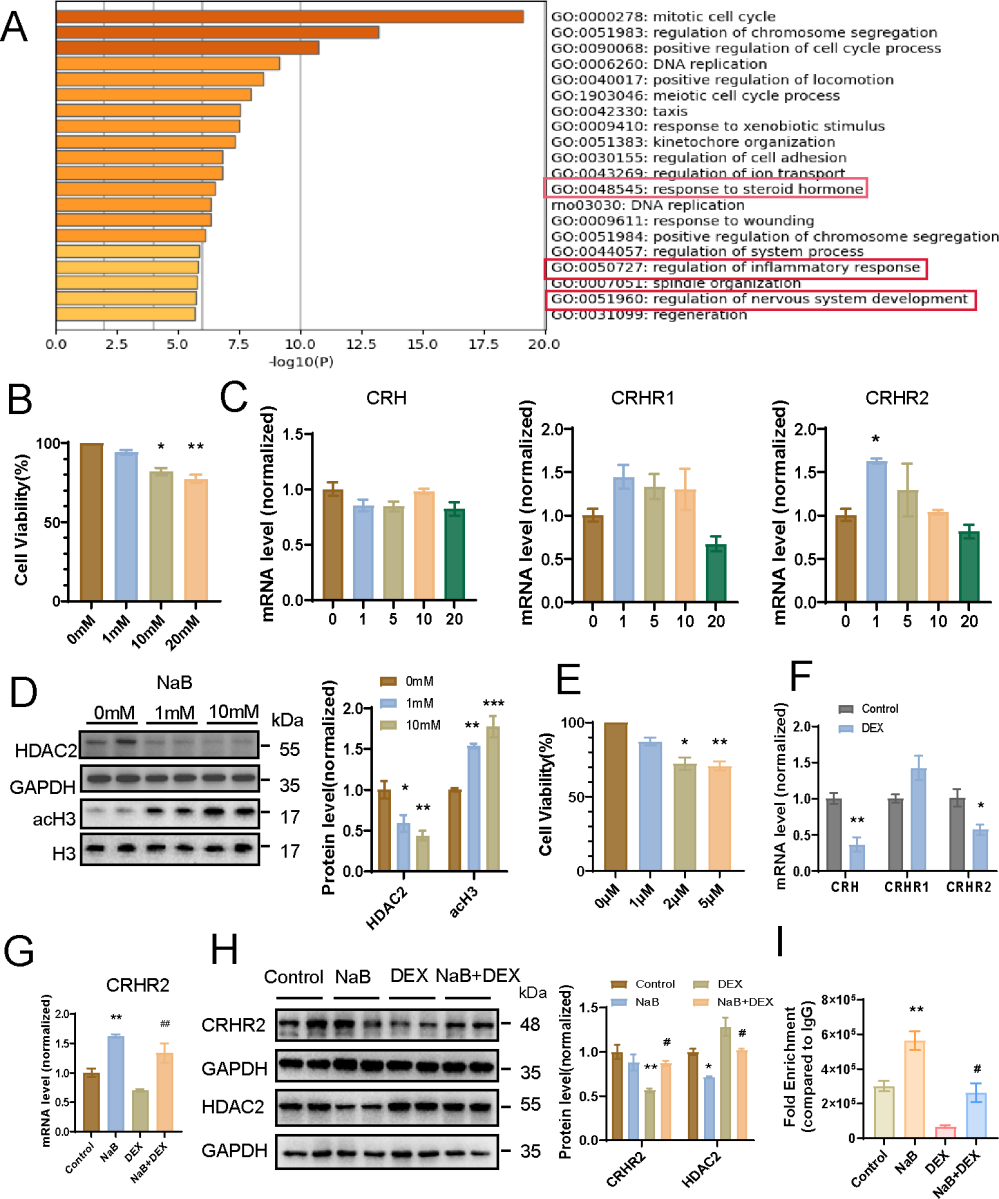
NaB and dexamethasone treatments regulate CRHR2 expression in PC12 cells. (A) GO enrichment analysis using gene set derived from GSE56516 (PC12 cells were exposed to NaB). (B) Cell Counting Kit-8 assay measuring PC12 cell viability treated with different concentrations of NaB (0–20 mM) for 24 hours (n = 3 per group, one-way ANOVA followed by Tukey post hoc test) C. CRH, CRHR1 and CRHR2 mRNA expression after exposure with different concentrations of NaB (0–20mM) in PC12 cells for 24 hours, as detected using qPCR and normalized to GAPDH [n = 3 per group, one-way analysis of variance (ANOVA) followed by Tukey post hoc test]. (D) Western blot and quantification analyses of HDAC2 and acH3 in PC12 cells treated with different concentrations of NaB (0–10 mM) for 24 hours (n = 3 per group, one-way ANOVA followed by Tukey post hoc test). (E) Cell Counting Kit-8 assay measuring PC12 cell viability treated with different concentrations of dexamethasone (0–5 μM) for 10 hours (n = 3 per group, one-way ANOVA followed by Tukey post hoc test). (F) CRH, CRHR1, and CRHR2 mRNA expression in PC12 cells after exposure with 1 µM dexamethasone for 10 hours, as detected using qPCR and normalized to GAPDH (n = 3 per group, two-tailed *t*-test). (G) CRHR2 mRNA expression in PC12 cells treated with control, NaB, dexamethasone, and NaB + dexamethasone, as detected using qPCR and normalized to GAPDH (n = 3 per group, two-tailed *t*-test). (H) Western blot and quantification analyses of CRHR2 and HDAC2 in PC12 cells treated with control, NaB, dexamethasone, and NaB + dexamethasone (n = 3 per group, two-way ANOVA followed by Tukey post hoc test). (I) ChIP–qPCR analyses of the enrichment of acetylated histone H3 on the promoter region of the CRHR2 gene in PC12 cells treated with control, NaB, dexamethasone, and NaB + dexamethasone (n = 6 per group, two-way ANOVA followed by Tukey post hoc test). Data are expressed as mean ± SEM. ***P < 0.001, **P < 0.01, *P < 0.05 vs control, ^##^P < 0.01, ^#^P < 0.05 vs DEX.

There are three differentiated types of PC12 cells, namely undifferentiated, poorly differentiated, and well-differentiated PC12 cells (22, 23). Currently, differentiated PC12 cells have been widely used in neurobiological studies with their neuronal features (23). Besides, the PC12 cells were also found to produce CRH (24). Thus, we used this cell line for further study *in vitro*. Microarray results about NaB- and vehicle-treated PC12 cells were acquired from NCBI-GEO repository (GSE56516). According to the differential genes derived from GSE56516, a total of 3,148 genes were significantly altered following treatment with NaB. We used the differential gene sets in the PC12 cells with or without NaB treatment for gene ontology (GO) enrichment by Metascape. The most highly represented GO items include response to steroid hormone, regulation of inflammatory response, and regulation of nervous system development (Fig. 4A). Additionally, items related to GC and corticosteroid were also included (Table S2). This enrichment analysis indicates that NaB treatment may regulate gene expression related to HPA axis in PC12 cells.

Then, CCK-8 assay was performed to determine whether NaB treatment had a cytotoxicity influence on PC12 cells. The cell viability analyses demonstrated that higher concentrations (10 or 20 mM) of NaB had a damaging impact on PC12 cells, while a lower concentration (1 mM) did not (Fig. 4B). As described above, CRHR2 expression level was found decreased in the hypothalamus of LPS-exposed offspring rats compared with control dams. We detected CRH and its receptors mRNA levels in PC12 cells exposed to different concentrations (0, 1, 5, 10, and 20 mM) of NaB for 24 hours. The result showed the CRHR2 mRNA level was enhanced by 1 mM NaB incubation (Fig. 4C). To further explore HDAC changes in the model of NaB-treated PC12 cells, we also analyzed the expression of HDAC2 and acetylated histone H3 by western blot, and the result showed that NaB exposure reduced HDAC2 and increased acetylated histone H3 expression levels in PC12 cells (Fig. 4D). It thus suggests NaB treatment might regulate CRHR2 expression via its HDAC inhibition role.

The viability of the PC12 cells following exposure to dexamethasone was also determined using a CCK-8 assay. Compared with the control group, higher concentrations of dexamethasone (2 and 5 µM) showed cytotoxicity to PC12 cells in 10 hours (Fig. 4E). In line with the previous study, our quantitative PCR analyses also showed the expression levels of CRH and CRHR2 were marked downregulated by 1 µM dexamethasone incubation for 10 hours (25) (Fig. 4F). Considering NaB and dexamethasone had opposite regulation roles on CRHR2 expression level, we wonder whether NaB could alleviate dexamethasone-induced CRHR2 downregulation via HDAC inhibition. Thus, PC12 cells were cultured and pre-exposed to NaB for 14 hours and then dexamethasone was added for a further 10 hours. Western blot was performed to analyze CRHR2 and HDAC2 expression levels in these cell groups. PC12 cells in the NaB + dexamethasone group significantly increased CRHR2 expression level in contrast to the dexamethasone group (Fig. 4H). Simultaneously, both in the NaB and NaB + dexamethasone groups, HDAC2 expression level was downregulated (Fig. 4H). To further reveal the involvement of acH3 in the regulation of CRHR2 gene, our chromatin immunoprecipitation (ChIP) assays indicated that acH3 enrichment on the CRHR2 promoter was significantly increased in the NaB + dexamethasone group compared with the dexamethasone group, implicating acH3 in the upregulated transcription of CRHR2 (Fig. 4I). These results demonstrate that higher histone acetylation level induced by NaB is responsible for CRHR2 restoration.

### NaB treatment rescued corticosterone and butyric acid abnormal changes in LPS-exposed offspring

**Fig 5:**
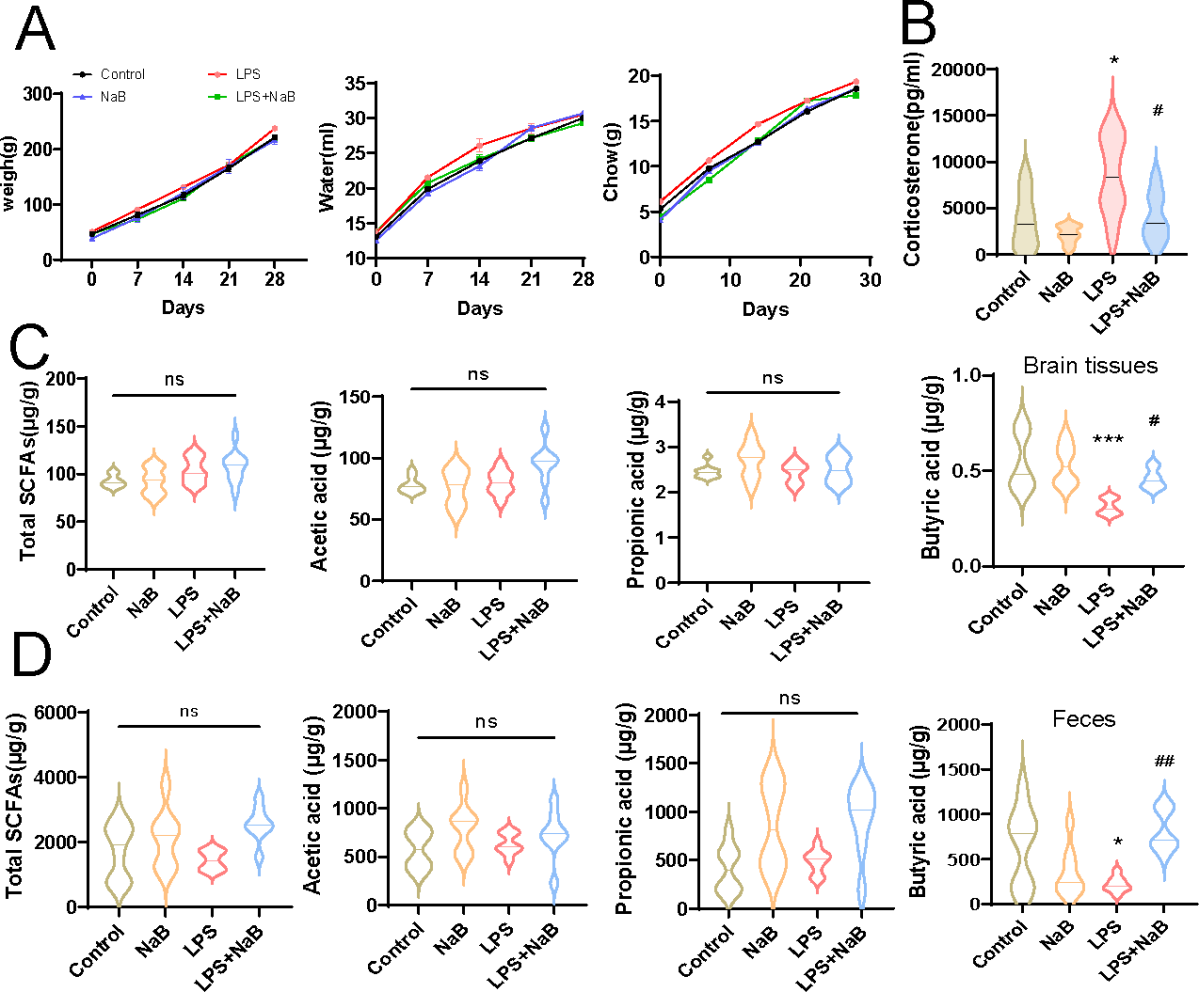
NaB treatment rescues corticosterone and SCFAs levels in LPS-exposed offspring. (A) Body weight gain, water, and chow consumption in the control, NaB, LPS, and LPS + NaB groups following NaB treatment for 28 days (two-way repeat-measures ANOVA, *n* = 8 per group). (B) Corticosterone level in serum of the control, NaB, LPS, and LPS + NaB groups (*n* = 8 per group, two-way ANOVA followed by Tukey post hoc test. (C) Total SCFAs levels, acetic, propionic, and butyric acid levels in the brain tissues of the control, NaB, LPS, and LPS + NaB groups (*n* = 8 per group, two-way ANOVA followed by Tukey post hoc test). (D) Total SCFAs levels, acetic, propionic, and butyric acid levels in the feces of the control, NaB, LPS, and LPS + NaB groups (*n* = 8 per group, two-way ANOVA followed by Tukey post hoc test). Data are expressed as mean ± SEM. ***P < 0.001, *P < 0.05 vs control; ^##^*P* < 0.01, ^#^*P* < 0.05 vs LPS; ns, not significant.

The finding that butyric acid concentration was decreased both in the feces and the brain tissues of LPS-exposed offspring prompted us to examine whether butyric acid supplement had beneficial effects on these offspring. During 4 weeks of 200 mM NaB administration, we found no significant differences on the body weight, water, and chow intakes in the control, NaB, LPS, and LPS + NaB groups, which suggests neither prenatal LPS administration nor postnatal NaB treatment affects nutrition condition and food consumption (Fig. 5A). Next, corticosterone level was measured within these groups; the results displayed that NaB treatment significantly decreased the serum corticosterone level of LPS-exposed rats (Fig. 5B). In addition, we also analyzed the SCFAs concentration in the brain tissues and the feces among them. Consistent with our findings in SCFAs described above, the total SCFAs, acetic and propionic acid levels were detected with no significant differences among them (Fig. 5C and D). After supplementing with NaB for 4 weeks, however, the butyric acid levels were significantly increased both in the brain tissues and the feces of LPS-exposed offspring (Fig. 5C and D). Together, these results suggest that NaB taken by LPS-exposed offspring normalizes its corticosterone and butyric acid concentrations.

### NaB treatment increased SCFA-producing bacteria in LPS-exposed offspring

**Fig 6:**
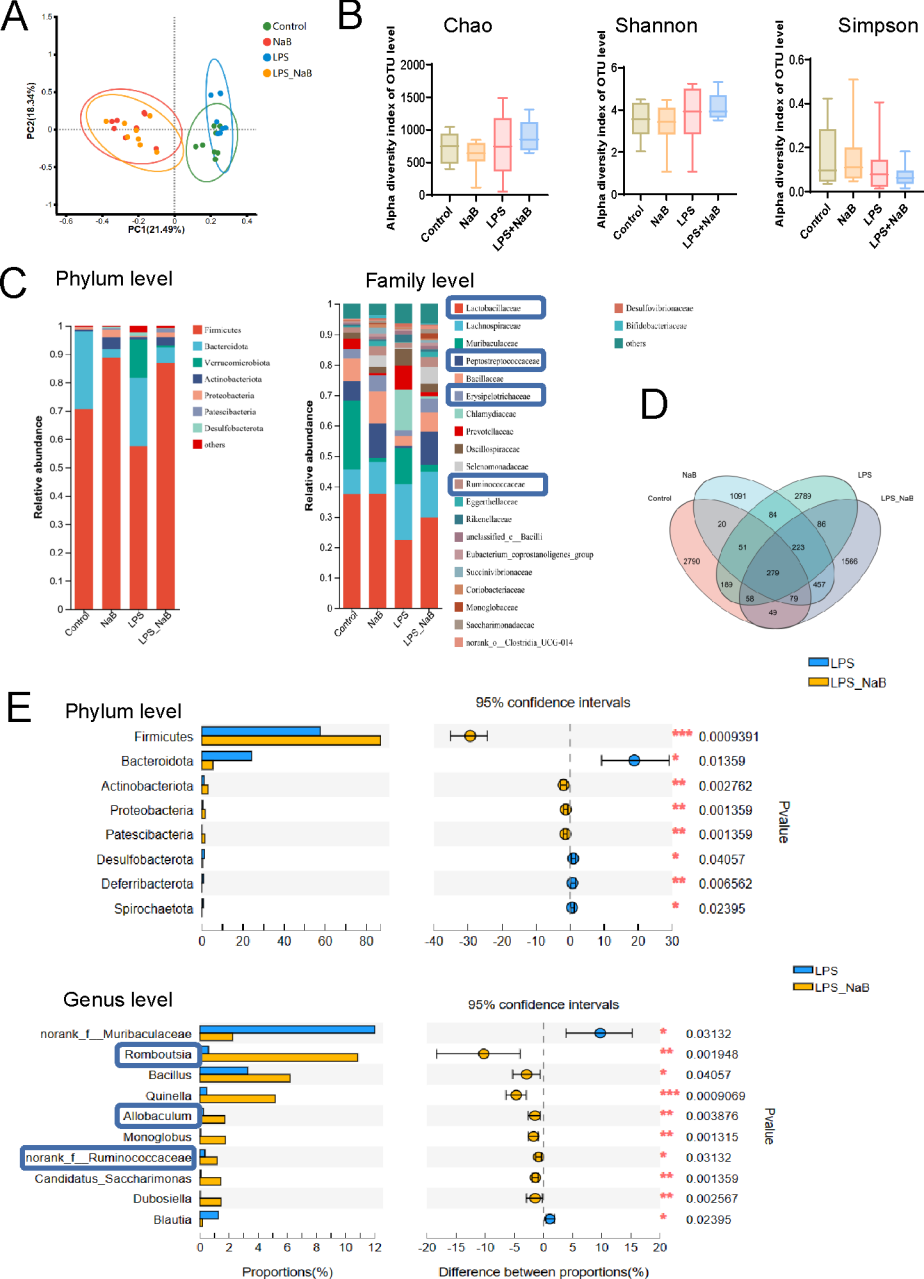
Changes in the gut microbiota composition among the control, NaB, LPS, and LPS + NaB groups. (A) PCoA plots of the microbiota composition among the control, NaB, LPS, and LPS + NaB groups at the ASV level (*n* = 8 per group). (B) Alpha diversity of the gut microbiota (Chao, Shannon, and Simpson indices) among the control, NaB, LPS, and LPS + NaB groups at the ASV level (*n* = 8 per group, Kruskal–Wallis H test followed by Tukey post hoc test). (C) Relative abundance of the bacterial taxa among the control, NaB, LPS, and LPS + NaB groups at the phylum and family levels. SCFA-producing bacteria are marked with blue frames. (*n* = 8 per group, Kruskal–Wallis H test followed by Tukey post hoc test). (D) Venn diagram depicting the number of bacterial taxa that were unique and shared among the control, NaB, LPS, and LPS + NaB groups at the ASV level. (E) Comparison of the dominant microbiota at the phylum and family levels between the LPS and LPS + NaB groups. SCFA-producing bacteria are marked with blue frames. (***P < 0.001, **P < 0.01, *P < 0.05, *n* = 8 per group, Wilcoxon rank sum test followed by Tukey post hoc test).

To further investigate the role of NaB administration on gut flora composition, we used 16S ribosomal RNA gene sequencing to detect microbiota changes in the control, NaB, LPS, and LPS + NaB groups. PCoA plot showed differences in microbial community structure after NaB administration both in the control and LPS-exposed offspring (Fig. 6A). However, no statistical differences were found in Chao, Shannon, and Simpson indices among the four groups (Fig. 6B). The compositions of the dominant microbiota at the phylum and family level were shown in Fig. 6C. *Lactobacillaceae,* one of the SCFA-producing bacteria, were the most abundant bacteria in the feces sample among them at family level. The Venn diagram plot of the gut flora indicated the distribution of amplicon sequence variants (ASVs) shared or distinguished with each other in these groups (Fig. 6D). Further analysis revealed that the abundance of *Firmicutes* was increased and *Bacteroidota* was decreased in the LPS + NaB group compared with the LPS group at the phylum level (Fig. 6E). Surprisingly, *Romboutsia*, *Allobaculum*, and *Ruminococcaceae*, which were found to produce SCFAs, were also increased at the genus level after NaB administration in LPS-exposed rats (26 - 28) (Fig. 6E). These findings suggest that NaB supplement could affect the gut microbiota profiles and improve SCFA-producing bacteria in LPS-exposed offspring.

### NaB treatment restored CRHR2 and histone acetylation expression levels and ameliorated autism-like behavior in LPS-exposed offspring

**Fig 7:**
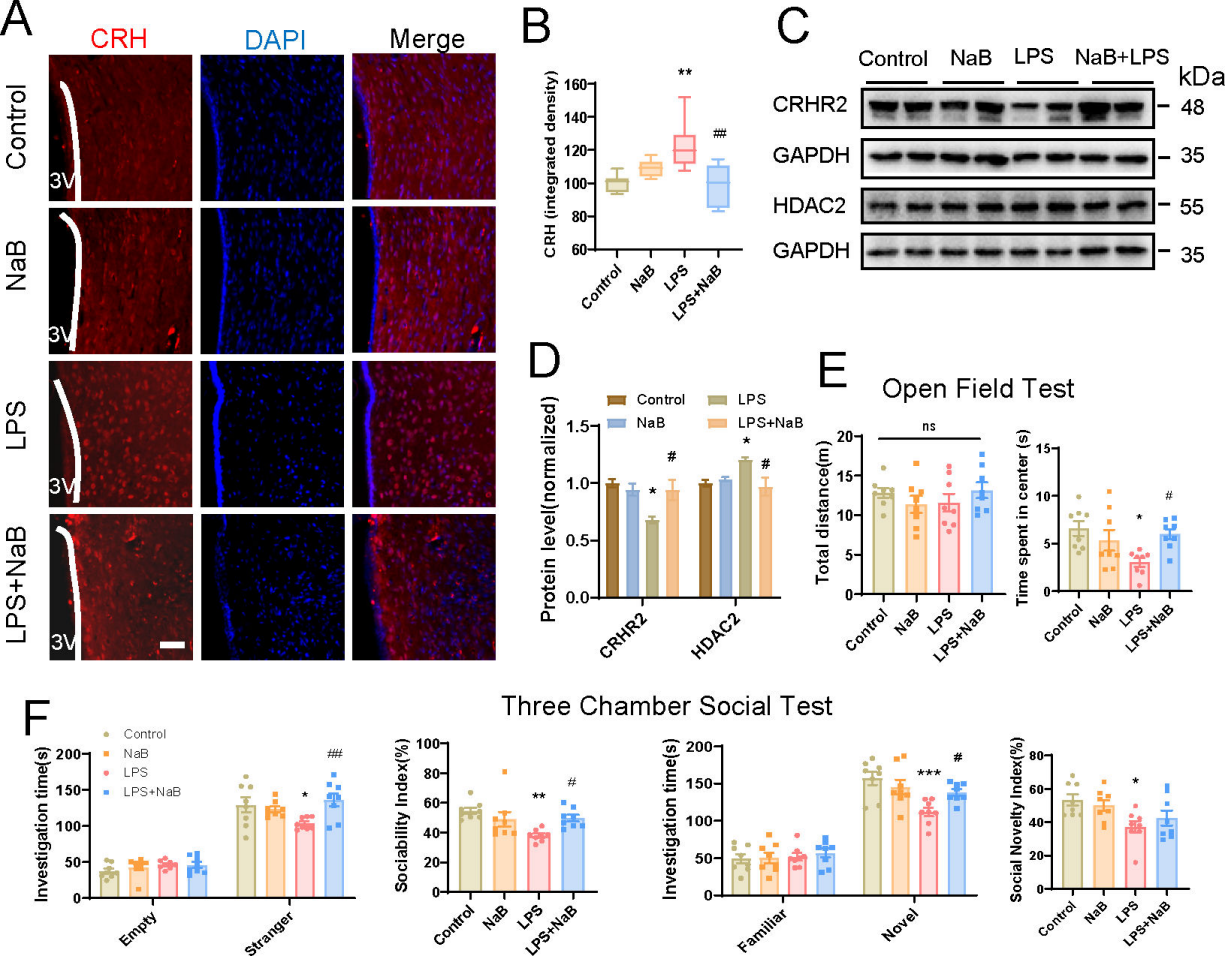
NaB treatment normalizes HPA axis and histone acetylation expression and ameliorates autism-like social deficits in LPS-exposed offspring. (A–B) Confocal images and quantification of CRH in hypothalamus slices of the control, NaB, LPS, and LPS + NaB groups (scale bars, 50 µM; 3V, third ventricle; *n* = 8 images from three rats per group, two-way ANOVA followed by Tukey post hoc test). (C–D) Western blot and quantification analyses of CRHR2 and HDAC2 in the hypothalamus from the control, NaB, LPS, and LPS + NaB groups (*n* = 3 per group, two-way ANOVA followed by Tukey post hoc test). (E) Total distance traveled and the time spent in center area during the open field test of the control, NaB, LPS, and LPS + NaB groups (*n* = 8 per group, two-way ANOVA followed by Tukey post hoc test). (F) The time spent in different chamber (A: Stranger rat vs B: Empty; or A: Novel rat vs B: Familiar rat) and sociability or social novelty index [Index = (A - B) / (A + B) * 100%] during the three-chamber social test of the control, NaB, LPS, and LPS + NaB groups (*n* = 8 per group, two-way ANOVA followed by Tukey post hoc test). Data are expressed as mean ± SEM. ***P < 0.001, *P < 0.05 vs control; ^##^*P* < 0.01, ^#^*P* < 0.05 vs LPS; ns, not significant.

Immunofluorescence staining showed that CRH expression in hypothalamus slices was elevated in the LPS group, and NaB supplement reduced its expression to the normal level (Fig. 7A and B). Western blot analyses revealed that NaB administration significantly increased CRHR2 expression level compared with those of the LPS-exposed offspring (Fig. 7D). Then, the HDAC2 expression level was also downregulated in the hypothalamus of the LPS + NaB group (Fig. 7D). Together, these results display that NaB treatment can normalize histone acetylation and CRHR2 expression level in the LPS-exposed offspring.

We further examined the impact of NaB on autism-related behavior in LPS offspring rats, which exhibited anxiety-like and social preference deficiency as described above. As shown in Fig. 7E, during the open field test, the LPS + NaB rats spent significantly more time in the center area than the LPS group, without alteration in locomotion. In sociability and social novelty test, the LPS + NaB group spent more time interacting with social and novel social stimuli than the LPS group (Fig. 7F). The sociability index and social novelty index were also analyzed, and a significantly elevated sociability index was detected in the LPS + NaB group (Fig. 7F). As for the social novelty index, though no significant difference was found between the LPS + NaB group and the LPS group, there was still an improvement tendency of social novelty in the LPS + NaB group (42.57 ± 4.543) compared with the LPS group (37.2 ± 3.543) (Fig. 7F). These results above demonstrate that NaB supplement can alleviate autism-like behavior in the LPS-exposed offspring rats.

## DISCUSSION

MIA offspring displayed aberrant anxiety and social behavior, cognitive and memory deficits, changes in immune and neuronal gene expression, and reduced dendritic spine density in the neonatal rats (29, 30). Both the ASD children and LPS-exposed ASD rats were found with alterations in SCFAs concentrations and GC levels, suggesting that LPS-exposed offspring exhibited the same phenotype as children with ASD. Decreased histone acetylation and increased corticosterone levels in LPS-exposed rats that we found suggested that maternal immune exposure may have a long effect on their offspring development. As reported, SCFAs can cross the blood-brain barrier (9). Thus, decreased butyric acid concentration both in the brain and feces of LPS-exposed rats showed that SCFAs (especially butyric acid) might have a critical role in the development of ASD. Consistent with our findings, SCFAs were also found altered in other ASD children and animal models. For example, Liu et al. found that acetic acid and butyric acid were decreased in children with ASD (8). Besides, acetic acid, butyric acid, and valeric acid were significantly decreased in the fecal sample of a valproic acid model of ASD compared to those in the control group (31). However, the role of SCFAs in ASD pathology remains controversial. Some clinical studies also reported no difference or even higher concentrations of SCFAs in ASD children (32, 33). These altered fecal SCFAs can be related to many factors, such as increased probiotic use, gut dysbiosis, impaired gut permeability, and so on (34). Thus, more studies are still needed to elucidate the role of SCFAs in ASD.

As for the HPA axis activation, we showed higher serum corticosterone levels in the LPS-exposed rats, which suggests excessive activation of the HPA axis. Prenatal exposure to LPS can cause inflammation damage in the offspring brain, and this inflammation can act as a crucial stress to activate the HPA axis directly (17). CRH is known to be an important coordinator of the HPA axis. When a stressful event happens, CRH can be released in the hypothalamus and triggers the downstream of the HPA axis to secrete cortisol. However, when the level of cortisol is overly elevated, CRH can be inhibited by cortisol through a negative feedback mechanism, which leads to the balance of the stress response (13). In addition, disturbances of the CRH system regulation could be related to many disorders, such as depression, anxiety, and inflammatory bowel diseases (35). Overactivation of HPA axis has been shown to impact social behavior and upregulation of CRH-expressing neurons in the PVN can induce social deficits in mice with normal microbiomes (19). The function of CRH peptide is mediated by two different GPCRs, CRHR1 and CRHR2. These two receptors share almost 70% amino acid homology but have different distributions in brain and peripheral tissues (36). CRHR2 plays a role in counteracting the effect of CRHR1 activation, which induces anxiety-like behavior and stress response (35). CRHR2-deficient mice also exhibit enhanced anxious behavior in contrast to mice lacking CRHR1 (37). Besides, a previous study revealed that prenatally stressed rats expressed lower CRHR2 mRNA in the amygdala (38). And thus, decreased CRHR2 expression level in the hypothalamus of our LPS-exposed offspring might also relate to its abnormal behavior.

A recent study demonstrated that maternal butyrate implementation rescued the social and partially repetitive behavior deficits in BTBR T^+^Itpr3^tf^/J (black and tan BRachyury) mice, a model of ASD (39). Apart from the maternal supplement, doses of NaB administered by intraperitoneal injections attenuated social deficits and enhanced memory in the passive avoidance of the BTBR mice themselves (40, 41). Chronic treatment with NaB also attenuates novel object recognition deficits and hippocampal dendritic spine loss in the valproic acid model of ASD (42). Previous studies also showed that butyric acid treatment actively modulated synaptic plasticity, induced broad neurotransmitter gene expression, and alleviated behavioral abnormalities (40, 43, 44). Besides, *Clostridium butyricum* (butyrate production microbes) is found to shape social dominance through modulating HDAC2 in the brain (45). Our 16S rRNA gene sequencing results showed that *Lactobacillus*, which could promote butyrate production, was the key differential abundant bacteria between the control and LPS-exposed rats at the family level. *Lactobacilli* are widely used in the production of fermented foods, such as dairy products, fermented vegetables, and so on (46). In addition, the *Lactobacilli* are an important component of microbiota in humans where they are colonized in the respiratory tract, gastrointestinal tract, urinary tract, and genital tract (46). For the digestive system, *Lactobacilli* can inhibit the translocation of pathogenic microbes and remodel the commensal microbiota composition to strengthen the intestinal barrier in the host (47). Furthermore, *Lactobacillus* species are also involved in Treg cell differentiation, which contributes to the host immune system and stress-induced social avoidance behavior (48). Of note, *Lactobacillus reuteri* could rescue social deficits in many ASD models by modulating the vagus nerve as well as the oxytocinergic and dopaminergic signaling in the brain (49). Therefore, *Lactobacillus* might serve as an ideal probiotic method for improving social behavior in individuals with ASD.

Butyric acid is known as a fine-tuned endogenous HDACs inhibitor, and HDAC family proteins can cause silencing of gene expression via condensing the chromatin architecture and altered histone acetylation (12, 50). Dysregulation of histone modification is involved in various types of cognitive deficits and social communication (51). In our study, downregulated histone acetylation level in LPS-exposed offspring and increased binding between acH3 and the promoter of CRHR2 in PC12 cells after NaB treatment also suggested an epigenetic mechanism in HPA axis regulation. Apart from CRHR2, epigenetic regulation exerts an important role in HPA axis homeostasis. For example, early life stress affects DNA methylation of the CRH gene promoter and the DNA methylation shows a negative correlation with CRH mRNA levels in the central amygdala (52). Similarly, early life stress also increased histone 3 lysine 9 acetylation levels at the GC receptor (GR) promoter in the amygdala of adult female rats (53). Moreover, decreased histone acetylation with increased HDAC5 levels was found at the GR exon I_7_ promoter in a model of depression-like behavior (54). Thus, HPA axis abnormality has been implicated in many neurological diseases, and detailed mechanisms underlying the effect of HPA axis on social behaviors await to be further studied.

Indeed, our study found NaB supplement after weaning could rescue social preference in the LPS-induced ASD-like rat model. However, only male rats were statistically analyzed in our study. Further research needs to be conducted on female rats. Additionally, our research verified the combination of CRHR2 promoter and acH3 *in vitro* but not *in vivo*; thus, whether SCFAs improve social behavior directly through the HPA axis is not proven and germ-free rats will provide stronger evidence. Besides, more studies focused on the hypothalamus for assessment of SCFAs concentration changes are still needed.

In summary, our study indicates that butyric acid has profound effects on social behavior, and an interesting mechanism on its histone acetylation regulation of HPA axis is raised to play important roles. LPS-exposed rats displayed lower histone acetylation as well as altered CRHR2 expression level. Moreover, increased acH3 binding to CRHR2 promoter after treatment with NaB *in vitro* suggests that histone acetylation influenced HPA axis regulation. Finally, supplementation of NaB rescued the social deficits, a core symptom of ASD, and normalized corticosterone concentration of LPS-exposed offspring.

## MATERIALS AND METHODS

### Study participants

All participants and their guardians were given a complete description of the study and informed consent was provided by the primary caregiver. The inclusion criteria were a diagnosis of ASD made by a developmental pediatrician through a series of structured interviews according to the *Diagnostic and Statistical Manual of Mental Disorders (Fifth Edition, DSM-5)* criteria. The exclusion criteria included other developmental disorders, neurological or psychiatric diseases, genetic metabolic disease, and a recent history of infection or antibiotic/probiotic use. The protocol was approved by the Medical Ethics Committee of Children’s Hospital of Chongqing Medical University, Approval Number: (2019) IRB (STUDY) No. 38. In total, 84 children with autism and 83 healthy controls (TD children) matched for age and gender participated in this study. Based on clinical available records, the diagnosis of autism was made by two developmental pediatricians at the Children’s Hospital of Chongqing Medical University through a series of structured interviews and met the criteria from the DSM-5.

### Animals and experimental design

Adult Sprague–Dawley rats were obtained from the Animal Care Center of Chongqing Medical University (Chongqing, China) and were housed under specific pathogen-free conditions. All experimental procedures were approved by the Ethics Committee of Children’s Hospital of Chongqing Medical University (IACUC Issue No: CHCMU-IACUC20220429010). Rats were mated and the presence of a vaginal plug was determined as E0.5. Then, a single dose of 100 mg/kg LPS (Sigma-Aldrich) or saline was randomly administered by intraperitoneal injection on E9.5. Day of birth was recorded as D0 and the offspring used for all experiments in this study were males. In the SCFAs intake experiment, NaB (Sigma-Aldrich, 303410) or saline was dissolved in drinking water (200 mM). This concentration was widely used in previous studies (51). We measured the volume of drinking water every day and water intake of the animals were controlled. Subsequently, all animals were randomly assigned to various experimental groups at D21: the saline (control) group, NaB group, LPS group, and LPS + NaB group. Dietary treatments were administered for 4 weeks, drinking water was refreshed two or three times per week. Offspring rats aged approximately 8 weeks were anesthetized by 1% pentobarbital sodium and sacrificed. Then, serum, feces, and the brain samples were collected from each group and analyzed.

### SCFA quantification by gas chromatography–mass spectrometry analysis

The feces samples of children with autism and healthy controls or brain tissues and feces samples of rats in the control, NaB, LPS, and LPS + NaB groups were collected. Acetic, propionic, isobutyric, butyric, isovaleric, valeric, hexanoic, heptanoic, octanoic, nonanoic, and decanoic acid levels were detected and analyzed. Briefly, metabolites were extracted by homogenizing and then centrifuged for 20 minutes at 5,000 rpm, 4°C. Transferring the supernatant into EP tubes and adding 0.1 mL 50% H_2_SO_4_ and 0.8 mL of extracting solution (25 mg/L stock in methyl tert-butyl ether) as internal standard. After centrifuging again, the supernatant was injected and detected by gas chromatography–mass spectrometry (SHIMADZU GC2030-QP2020 NX). The system utilized a HP-FFAP capillary column (J&W Scientific, Folsom, CA, USA). The mass spectrometry data were acquired in Scan/SIM mode with the m/z range of 33–150 after a solvent delay of 3.5 minutes. The recovery rate was used to perform quality control.

### Cortisol or corticosterone determination

Plasma samples from children and serum samples from offspring rats were collected. Cortisol and corticosterone levels were assayed using a commercially available Cortisol ELISA (enzyme-linked immunosorbent assay) Kit (No. 500360 Cayman Chemical Company, USA) and Corticosterone ELISA Kit (No. 501320 Cayman Chemical Company, USA) according to the manufacturer’s instructions. Briefly, ELISA standard and ELISA buffer were prepared and added in wells, then ELISA monoclonal antibody was added to sample wells or maximum binding wells. The plate was incubated overnight at 4℃ and rinsed five times. After adding 200 µL Ellman’s reagent, the plate was developed in the dark for 2 hours and detected at a wavelength between 405 and 420 nm. The concentration was determined by standard curve.

### Behavioral tests

Behavioral studies were conducted on the rats at 7 weeks of age for open field exploration and social interaction as our previous study described (55). In brief, open field test was performed using a square apparatus (50 cm × 50 cm). The rats were placed in the cage for 5 minutes, then the total distance traveled, the time spent in grooming or in the center zone area, and the number of entries were measured. The three-chamber social test was performed to assess sociability and social novelty. The first phase featured an age- and sex-matched stranger rat (A) and an empty cage (B) in the two side chambers, and the second phase contained a stranger rat (A’) and a familiar rat (the stranger rat in the first phase, B’) in the two side chambers. Based on the amount of time spent in each chamber, the sociability index and the social novelty index were calculated according to the following formulas: Index = (A − B) / (A + B) * 100%. All parameters were automatically recorded using the ANY-Maze Video Tracking System (ANY-Maze, USA).

### 16S rRNA gene sequencing

The feces samples of rats in the control, NaB, LPS, and LPS + NaB groups were collected. Total genomic DNA was extracted from the fecal DNA kit (QIAamp DNA Stool Mini Kit) according to the manufacture’s protocol. DNA concentration and purity were monitored on 1.0% agarose gels. The V3–V4 of the bacterial 16S rRNA gene were amplified by a PCR system (GeneAmp 9700, ABI, USA) with primer pairs 338F (5′-ACTCCTACGGGAGGCAGCAG-3′) and 806R (5′-GGACTACHVGGGTWTCTAAT-3′). The PCR product was purified using the AxyPrep DNA Gel Extraction Kit (Axygen Biosciences, Union City, CA, USA) and quantified using Quantus Fluorometer (Promega, USA) according to manufacturer’s instructions. Purified amplicons were pooled in equimolar amounts and paired-end sequenced on an Illumina MiSeq PE300 platform (Illumina, San Diego, CA, USA) according to the standard protocols by Majorbio Bio-Pharm Technology (Shanghai, China). Raw sequencing reads were de-multiplexed and then quality-filtered by fastp (v0.19.6) and merged by FLASH (v1.2.11). Then the high-quality sequences were de-noised using DADA2 plugin in the Qiime2 (v2020.2) pipeline. DADA2 de-noised sequences are usually called ASVs. Alpha diversity analyses (Chao, Shannon, and Simpson indices) were performed with Mothur (v1.30.1). For beta diversity analysis, PCoA based on Bray–Curtis distances was calculated using vegan R package (v 2.5–3). SCFA-producing bacteria were identified according to previous research (21, 26 - 28).

### Western blotting

Total protein in the hypothalamus of rats and PC12 cell lysates were extracted according to the manufacturer’s protocols (Cat number: KGP3100; KeyGEN BioTECH). The proteins were separated by electrophoresis (Bio-Rad, CA, USA) and transferred onto 0.45 µM PVDF (polyvinylidene fluoride) membranes. After blocking, the membranes were incubated with antibodies at 4°C overnight and secondary antibodies for 1.5 hours at room temperature. The primary antibodies used for western blotting were as follows: CRH (1:1,000, Proteintech, 10944–1-AP), CRHR1 (1:1,000, abcam, ab77686), CRHR2 (1:1,000, abcam, ab236982), HDAC1 (1:1,000, HuaBio, ET1605-35), HDAC2 (1:1,000, HuaBio, ET1607-78), HDAC3 (1:1,000, HuaBio, ET1610-5), HDAC8 (1:1,000, HuaBio, ET1612-90), acH3 (1:1,000, Sigma, 06599), Histone 3 (H3) (1:1,000, Abways, CY6587), and GAPDH (1:5,000, Proteintech, HRP60004).

### Real-time PCR

Total RNA in the hypothalamus of rats and PC12 cell lysates were extracted according to the RNA Extraction Kit (LS1040, Promega). Prime Script RT Reagent Kit (Takara, Shiga, Japan) was used to generate cDNA from mRNA. Real-time PCR was performed using the SYBR-Green Real-time PCR Kit (Takara) and a CFX96 real-time PCR detection system (Bio-Rad, USA). The mRNA expressions were calculated and analyzed by 2^−ΔΔCT^ formula and normalized to GAPDH.

### RNA sequencing and bioinformatics analysis

Gene expression data from microarray study of PC12 treated with different concentrations of NaB (GSE56516 from GPL1355) were downloaded from the Gene Expression Omnibus (https://www.ncbi.nlm.nih.gov/geo/); GSM1363116 (Control PC12 cells) and GSM1363118 (6 mM SB) were selected in this study.

### Cell culture and CCK8 measurement

PC12 (rat pheochromocytoma) cells were grown in DMEM (Dulbecco's Minimal Essential Medium) (Gibco, Grand Island, USA) supplemented with 10% fetal bovine serum and 5% horse serum (Gibco, Grand Island, USA). The cells were grown in a humidified atmosphere of 5% CO_2_ at 37°C. The cell viability was measured by Cell Counting Kit-8 (CCK-8, Proteintech, China) assay according to the manufacturer’s instructions.

### ChIP and qPCR (ChIP-qPCR)

ChIP was performed using a ChIP kit (Abclonal, China), and the protein–DNA interaction in PC12 cells was fixed by crosslinking for 10 minutes with 1% formaldehyde, then cells were incubated for 5 minutes with glycine. For sonication, PC12 cells chromatin were sheared into 200–800 bp fragments by 15 minutes of medium-power sonication. The chromatin fragments were incubated with 4 µg of an anti-acH3 primary antibody (06599, Sigma) or a negative control IgG antibody for at least 4 hours at 4℃. ChIP signals were quantified as fold enrichment using the comparative ^ΔΔ^Ct method. Primers for rat CRHR2 promoter were designed as follows: sense: 5′–AGTGATTGGACCACACCCAC–3′, antisense: 5′–GGCTCTTTAAACCGTCCCGT-3′.

### Immunofluorescence staining

Rats were perfused with 4% paraformaldehyde and then dehydrated with 30% sucrose. Brain slices with a thickness of 30 µM were permeabilized and blocked with 5% BSA (bovine serum albumin) in 0.3% Triton X-100 containing PBS (phosphate-buffered saline). Subsequently, the slices were incubated with CRH antibody (1:1,000, Proteintech, 10944–1-AP) at 4°C for 24 hours. The secondary antibodies in the blocking solution were applied, followed by nucleus staining with DAPI (4,6-diamino-2-phenyl indole). The slices were photographed under a fluorescent microscope (Nikon, Tokyo, Japan) and analyzed by ImageJ software.

### Statistical analysis

All data were shown as the mean with standard error of the mean (mean ± SEM) using SPSS (v.20) and Graphpad Version 9.0 (GraphPad Software Inc., USA). Data were performed using one-way and two-way ANOVA, Wilcoxon rank sum test, and Kruskal–Wallis H test with Tukey post hoc analysis or unpaired Student’s *t*-test unless otherwise specified. The Shapiro–Wilk test was used for normality test. GO functional enrichment analysis was carried out by Metascape (http://www.metascape.org/gp/index.html). Probabilities of *P <* 0.05 were considered significant in figures denoting *P*-values as follows: *P < 0.05, **P < 0.01, ***P < 0.001, ****P < 0.0001 versus control and ^#^*P <* 0.05, ^##^*P* < 0.01, ^###^*P <* 0.001 versus LPS or dexamethasone.

## Data Availability

Raw sequence data of the microbiota that support the findings in our research have been deposited into NCBI’s Sequence Read Archive under accession number PRJNA970966.

## SUPPLEMENTAL MATERIAL

TABLE S1 msystems.00415-23-s0001.xlsx SCFAs measurement results for feces samples of children and feces or brain samples of rats.

TABLE S2 msystems.00415-23-s0002.xlsx Glucocorticoid and corticosterone GO items and genes using gene set derived from GSE56516 (PC12 cells were exposed to sodium butyrate).

ASM does not own the copyrights to Supplemental Material that may be linked to, or accessed through, an article. The authors have granted ASM a non-exclusive, world-wide license to publish the Supplemental Material files. Please contact the corresponding author directly for reuse.
