# Erratum for Wang et al., “Sodium butyrate facilitates CRHR2 expression to alleviate HPA axis hyperactivity in autism-like rats induced by prenatal lipopolysaccharides through histone deacetylase inhibition”

**DOI:** 10.1128/msystems.00915-23

**Published:** 2023-10-10

**Authors:** Xinyuan Wang, Zhujun Sun, Ting Yang, Fang Lin, Shasha Ye, Junyan Yan, Tingyu Li, Jie Chen

## ERRATUM

Volume 8, no. 4, e00415-23, 2023, https://doi.org/10.1128/msystems.00415-23. Page 16, Acknowledgments, “82372592” should read “82372559.”

Page 17, Funding, “82372592” should read “82372559.”

